# Autologous bone graft versus demineralized bone matrix in internal fixation of ununited long bones

**DOI:** 10.1186/1752-2897-3-11

**Published:** 2009-12-15

**Authors:** Oliver Pieske, Alexandra Wittmann, Johannes Zaspel, Thomas Löffler, Bianka Rubenbauer, Heiko Trentzsch, Stefan Piltz

**Affiliations:** 1 Department of Trauma Surgery, Campus Grosshadern, University Hospital of Munich, Germany

## Abstract

**Background:**

Non-unions are severe complications in orthopaedic trauma care and occur in 10% of all fractures. The golden standard for the treatment of ununited fractures includes open reduction and internal fixation (ORIF) as well as augmentation with autologous-bone-grafting. However, there is morbidity associated with the bone-graft donor site and some patients offer limited quantity or quality of autologous-bone graft material. Since allogene bone-grafts are introduced on the market, this comparative study aims to evaluate healing characteristics of ununited bones treated with ORIF combined with either iliac-crest-autologous-bone-grafting (ICABG) or demineralized-bone-matrix (DBM).

**Methods and results:**

From 2000 to 2006 out of sixty-two consecutive patients with non-unions presenting at our Level I Trauma Center, twenty patients had ununited diaphyseal fractures of long bones and were treated by ORIF combined either by ICABG- (n = 10) or DBM-augmentation (n = 10). At the time of index-operation, patients of the DBM-group had a higher level of comorbidity (ASA-value: p = 0.014). Mean duration of follow-up was 56.6 months (ICABG-group) and 41.2 months (DBM-group). All patients were clinically and radiographically assessed and adverse effects related to bone grafting were documented. The results showed that two non-unions augmented with ICABG failed osseous healing (20%) whereas all non-unions grafted by DBM showed successful consolidation during the first year after the index operation (p = 0.146). No early complications were documented in both groups but two patients of the ICABG-group suffered long-term problems at the donor site (20%) (p = 0.146). Pain intensity were comparable in both groups (p = 0.326). However, patients treated with DBM were more satisfied with the surgical procedure (p = 0.031).

**Conclusion:**

With the use of DBM, the costs for augmentation of the non-union-site are more expensive compared to ICABG (calculated difference: 160 €/case). Nevertheless, this study demonstrated that the application of DBM compared to ICABG led to an advanced outcome in the treatment of non-unions and simultaneously to a decreased quantity of adverse effects. Therefore we conclude that DBM should be offered as an alternative to ICABG, in particular to patients with elevated comorbidity and those with limited availability or reduced quality of autologous-bone graft material.

## Introduction

The development of non-unions depends on several factors, such as energy-level of trauma, type of fracture, soft tissue involvement, type of applied treatment, and various endogenous factors [1-3]. According to literature, non-union will occur in approximately 10% of fractures after conservative or operative treatment [4]. The use of iliac crest autologous bone graft (ICABG) is widely considered as gold standard for a number of reasons, including osteogenic, osteoconductive, and osteoinductive properties and the lack of disease transmission or of immunogenicity [5-7]. However, the use of autograft may be at risk of major drawbacks, such as limited availability and variable quality of the graft, hematoma, infection, increased operative time and bleeding, chronic donor site pain, and additional cost [8-15]. Subsequently, research has focused on the development of novel bone graft substitutes for the last decades [16,17].

In 1965, Urist at al. first described an osteoinductive substance while preparing soluble extracts from demineralized bone [18]. Since this pioneering work, a large body of data obtained by preclinical animal studies has supported the utility of demineralized bone matrix (DBM) in human clinical settings. Nevertheless, there still is a lack of clinical studies: A recent MEDLINE search using the term "demineralized bone matrix" restricted to "clinical study" and "English language" demonstrated, that only four references were found that were dealing with DBM-treatment in cases of a non-union of long bones [5,19-22]. Therefore, De Long et al. concluded that evidence for or against the use of DBM is still at a low level (Level-IV or V studies with consistent findings) [17].

In 2000, DBM Grafton^® ^(Osteotech Inc., Eatontown, NJ, USA) was introduced in our Department of Trauma Surgery as an alternative to iliac crest autologous bone graft (ICABG), particularly for morbid patients or those with decreased quantity or quality of autologous bone graft. Therefore, the aim of the present study was to report our experience in augmenting non-unions either with DBM or ICABG.

## Methods

### Patients

All patients presenting to our Level I trauma centre during a seven year period (01/2000 - 12/2006) with ununited fractures of upper and lower extremities' long bones were retrospectively selected for the study. Non-union was defined as the lack of bone healing by at least six months after fracture. For analysis, we compared those patients who had had ORIF and augmentation with ICABG ("autologous-group") and those with the use of demineralized bone matrix (DBM Grafton^®^; Osteotech, Eatontown, New Jersey) ("allograft-group"). Further inclusion criteria were: (1) patient over 18 years; (2) atrophic and diaphyseal non-union; (3) no segmental defect; (4) closed fracture or open I° according to the Gustilo-classification at initial presentation [23] and no clinical, radiographic, or laboratory evidence of infection; (5) not more than one previous operation at the non-union site; and (6) a minimum of 12 months of follow-up after index operation. We excluded patients fulfilling any of the following criteria: fractures secondary to a malignant tumor, immunosuppressive therapy, severe systemic disease or history of reflex sympathetic dystrophy and patients receiving other augmentation types than either ICABG or DBM.

During the observational period sixty-two consecutive patients with ununited long bone fractures were identified. Of these patients, two subjects were treated conservatively, twenty-two victims had intraarticular fractures, fourteen met at least one exclusion criterion and four patients were operated by using an autograft and allograft composite and therefore had to be excluded, because they could not be assigned for one of the two study groups. Thus 10 patients were identified in the autologous- and 10 patients in the allograft-group.

### Surgical Procedure

All surgical procedures were performed under the supervision of experienced orthopaedic trauma surgeons and followed a specific operation-protocol: All patients obtained a single shot antibiotic (1.5 g cefuroxime i.v.) straight preoperatively and underwent general anaesthesia. After exposure of the non-union site, several specimens for microbiological cultures were obtained. In case of prior fracture-instrumentation, implants were removed. Thereafter, non-union site was radically debrided of intervening scar tissue and sclerotic bone fragments with preservation of muscle and soft-tissue attachments to avoid devascularization. In addition, the medullary canal was opened both proximally and distally to complete the freshening of the bone ends. The non-union site was then reduced adequately by gently impacting the distal into the proximal fragment to obtain osseous contact and thereafter prepared for bone-grafting. The selection of the bone-graft-type, either ICABG or DBM (DBM Grafton^® ^Putty 2.5 cc; Osteotech, Eatontown, New Jersey), was based on the experience of the surgeon in charge. The graft was placed into the medullary canal and around the nonunion site. Finally, the bone ends were instrumented to achieve stable fixation of the ununited fracture. Intraoperative anteroposterior and lateral radiographs were used to confirm adequate placement of hardware and bone alignment.

### Follow-up

After hospitalization for the index-operation, all patients were clinically and radiographically investigated at our orthopaedic trauma outpatient department until the end of the non-union-related treatment. For the purpose of this study, follow-up was performed at least twelve months after the index-operation. Data assessment was performed by one investigator, who was not involved in the non-union treatment of the patients (A.W.). Blinding of the investigator was obviously not possible since all patients treated by ICABG were easily identifiable by the scar at the iliac crest donor site.

All patients completed the standardized baseline and follow-up questionnaire to obtain general information about the baseline data as well as the course of fracture treatment and non-union healing. Thereafter, the standardized telephone-interview was performed by the investigator to clarify the data. Sex, age, ASA value (American Society of Anaesthesiologists classification), body-mass-index (BMI), smoking status, time from trauma to index-operation and time from index-operation to follow-up were documented. Additionally, all postoperative complications related to the index-operation were documented. Furthermore, intensity of persistent pain at the prior ununited fracture site as well as the donor site in ICABG-patients was recorded by the use of a numeric rating scale (NRS) ranging from 0 (no pain) to 10 (maximum of pain). Clinical healing was defined as full weight bearing or complete function. Osseous healing was defined as a radiologically complete bridging callus formation with crossing trabeculae on anteroposterior and lateral radiographs obtained during the study period. All x-rays were investigated by study-independent radiologists. Furthermore, the level of patient's dissatisfaction concerning the non-union surgical procedure was documented with the use of a numeric rating scale (NRS) (range 0 to 5) by the following specifications: (0) satisfied, (1) minimal dissatisfied, (2) marginal dissatisfied, (3) partial dissatisfied, (4) mostly dissatisfied, and (5) extremely dissatisfied.

In case of insufficient quality of outcome data obtained by the follow-up questionnaire and the telephone-interview, patients were evaluated by the investigator clinically and x-rays of the surgical site were taken if necessary. For analysis, patient's personal data were anonymized.

### Statistical Analysis

For the comparison of both study groups (autologous-group and allograft-group) the Mann-Whitney *U *test was used to evaluate the differences with regard to the demographic and follow-up data. The Spearman rank correlation coefficient was used to evaluate the association between various factors (patient's age, sex, the American Society of Anaesthesiologists classification, BMI, prior fracture treatment, as well as smoking behaviour) and outcome after index-operation. Level of significance was set at p ≤ 0.05.

## Results

As shown in table 1, no differences were documented between the autologous- and allograft-group concerning demographic data, BMI, smoking behaviour, location of non-union, prior instrumentation at non-union site, and mean time from ununited fracture to index-surgery. ASA values were significantly higher in the allograft-group, indicating that these patients had more comorbidities at the time of surgery (p = 0.014). In both groups, comparable types of implants were used for fixation of united fracture during index-surgery (p = 0.255).

**Table 1:** Baseline data in patients with ununited long bone fractures treated by ORIF and augmentation with either autograft (n = 10) or allograft (n = 10).

		autograft group	allograft group	p-value Mann-Whitney-Test
**Sex**	[n]			0.383
	male	6	4	
	female	4	6	
**Age**	[years]			0.289
	mean	50.6	57.9	
	range	27-81	31-85	
**Body-mass-index**	[m/kg^2^]			0.450
	mean	25.0	24.5	
	range	19.2-28.4	18.8-39.0	
**Smoking-Status**	[package-yrs]			0.466
	mean	1.2	5.0	
	range	0-12	0-30	
**Location of non-union**	[n]			0.420
	upper arm	2	4	
	forearm	5	2	
	femur	0	3	
	lower leg	3	1	
**Fracture to index surgery**	[months]			0.183
	mean	8.7	14.6	
	range	6-16	6-54	
**Prior instrumentation**	[n]			0.654
	none	2	5	
	plate	7	2	
	intramedullary device	1	3	
**ASA Score**				0.014
	mean	1.4	2.1	
	range	1-2	1-3	

### Follow-up

**Figure 1:**
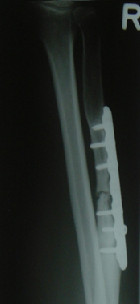
**X-ray of the right forearm 19 months after ORIF of a 34 year old man showing a persistent non-union of the ulna**.

**Figure 2:**
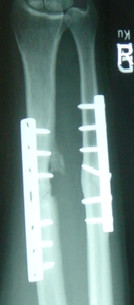
**X-ray of the right forearm 12 months after ORIF of a 57 year old man showing a persistent non-union of the radius**.

As shown in table 2, the mean follow-up time was 56.6 months (range 18-87 months) in the autologous and 41.2 months (range 12-69 months) in the allograft-group (p = 0.240). The mean time for clinical healing was comparable in both groups (p = 0.168) as well as for radiological consolidation (p = 0.327). Nevertheless, there was a lack of bone bridging in two patients treated by ICABG (autologous healing-rate: 80%) whereas all ununited fractures treated by DBM showed completed bone healing during the study period (allograft-healing-rate: 100%) (p = 0.146). Both patients of the autograft-group who failed to heal after the index-operation had a persistent non-union located at the forearm: One patient was a 34 year old male non-smoker who had to be re-operated 19 months after the index-operation because of pain, reduction of arm-function and implant failure at the ulna (see figure 1). The second ICABG-treated patient with impaired consolidation was a 57 year old male non-smoker. His x-rays twelve months after the index operation showed a stiff non-union with a good alignment and no radiological signs for implant loosening. Since he had no pain, no decreased arm-function during daily life activities and no reduction in his professional tasks as family doctor, he refused revision of the persistent non-union (see figure 2).

**Table 2:** Follow-up data in patients with ununited long bone fractures treated by ORIF and augmentation with either autograft (n = 10) or allograft (n = 10).

		autograft group	allograft group	p-value Mann-Whitney-Test
**Follow-up time**	[months]			0.240
	mean	56.6	41.2	
	range	18-87	12-69	
**Incidence of Bone Consolidation**	[n]	8	10	0.146
	%	(80%)	(100%)	
**Healing time: clinical**	[months]			0.168
	mean	8.3	4.1	
	range	2-24	2-8	
**Healing time: radiological**	[months]			0.327
	mean	10.9	11.9	
	range	2-40	2-21	
**Pain intensity: at rest**	[NRS]			0.326
	mean	2.0	1.1	
	range	0-6	0-5	
**Pain intensity: with physical activity**	[NRS]			0.936
	mean	2.3	2.2	
	range	0-7	0-6	
Level of treatment-dissatisfaction**	[NRS]			0.031
	mean	2.9	1.4	
	range	0-5	0-2	

* Pain intensity at follow-up were documented by the Numeric Rating Scale with a range from 0 (no pain) to 10 (maximum pain).

**Level of dissatisfaction concerning non-union surgical procedure at follow-up were documented by the Numeric Rating Scale ranging from of 0 (satisfied) to 5 (extremely dissatisfied).

### Donor site complications

One obvious difference between the two groups was the additional surgery for iliac crest bone harvesting in the autologous-group. These patients showed a donor-site-related morbidity rate of 20% since one subject suffered permanent pain at the iliac crest (VAS 3) and another victim complained about disabling keloid-formation at the donor-site-scar, which was associated with moderate pain (VAS 2) (p = 0.146). None of the patients suffered harvesting-related swelling, redness, drainage, infection or neurological deficits.

### Intensity of pain and level of treatment-dissatisfaction

At follow-up, patients of the both groups stated to have approximately equal intensity of index-operation-related pain (at rest: p = 0.326; with physical activity: p = 0.936) (see table 2). Additionally, four patients of the autologous-group (40%) compared to all patients of the allograft-group (100%) were satisfied or only minimally dissatisfied with the non-union treatment. Thus, patients of the allograft-group were significantly less dissatisfied with the treatment compared to those of the autologous-group (p = 0.031) (see table 2).

## Discussion

The ideal bone graft substitute should provide three key elements: (1) osteogenetic cells to facilitate bone regeneration; (2) osteoinductive factors to induce bone formation; and (3) an osteoconductive matrix to directly stimulate bone deposition. Osteoconductive materials have no capability to form bone or induce its formation *per se*. They merely provide an interconnected biocompatible scaffold, which local osseous tissue can utilize to regenerate living bone. Osteoinductive materials facilitate new bone formation by allowing cells in the local environment to undergo phenotypic conversion to osteoprogenitor cell types capable of formation of bone. "Osteogenic" is a graft material that has the inherent capacity to form bone, which implies that it has cells such as osteoblasts or osteocytes, capable of producing bone [2,4-7,16,17,24].

In this context, the best available alternative to autologous bone grafting is the use of an allograft. However, currently available allograft DBM formulations may differ considerably with regard to their bone inductive activity, mainly dependent on biological properties of the graft and the methods of allograft preparation [24]. DBM Grafton^® ^(Osteotech Inc., Eatontown, NJ, USA), which was used in this study as bone substitute in the allograft-group, is a type of processed allograft bone in combination with glycerin. As shown in animal studies, DBM Grafton^® ^has osteoconductive and osteoinductive potential: histologically, new bone formation could be shown after DBM-application [25,26].

Only few clinical studies are published to demonstrate DBM-efficacy as bone substitute and even less reports documented the outcome of DBM used in the treatment of long bone non-unions [5,17]. In 2003, Wilkens et al. published data using an injectable type of DBM called "AlloMatrix Injectable Putty": 30 of 35 patients with non-union in multiple bone types went on to union in an average of 3.5 months [21]. The same author showed that the percutaneous use of a mixture of autologous bone marrow and allograft DBM (AlloMatrix) led in 61 of 69 patients with stiff non-unions of long bones to union in an average period of 8.1 months [22]. Unfortunately, both studies were performed without a control group leading to a decreased Level of Evidence [5]. In 2005, Ziran et al. reported data of a retrospective comparative study using cancellous bone chips combined either with DBM Grafton^® ^(n = 25) or with DBM Orthoblast (n = 13) for the treatment of non-unions or impending non-unions in heavy smokers: Healing on the first graft attempt was observed in 52% of the DBM Grafton^® ^and 85% of the DBM Orthoblast group [20]. Since this study used DBM in combination with another graft-type, the question kept unanswered, in what extent the observed results were influenced by DBM. In 2006, Hierholzer et al. published a retrospective consecutive cohort study of ununited diaphyseal fractures of the humerus. Cases were treated with open reduction and internal fixation using a reconstruction plate and either iliac crest bone graft (n = 45) or DBM Grafton^® ^(n = 43). In the iliac crest bone group, clinical and radiological union was achieved in 100% in an average of 4.5 months compared to 97% in the Grafton^® ^group in 4.2 months [19]. One limitation of that study might be that the results are applicable only for the diaphyseal humerus non-unions, since DBM was used only in this specific long bone.

The aim of the present study was to evaluate the effect of DBM compared to ICABG in the treatment of non-unions of extraarticular long-bones in the upper and lower extremities. Since patients' baseline data as well as the observed radiological and pain intensity outcome during follow-up was comparable in both groups our study could demonstrate, that DBM has the same biological efficacy in promoting bone healing of non-unions compared to ICABG. Nevertheless, 20% of the autologous-group suffered considerable long term donor site complications at the iliac crest region. Additionally, patients treated by ICABG claimed elevated dissatisfaction concerning the non-union surgical procedure compared to those of the allograft-group during follow-up.

Since it is well known, that the biological potential of bone healing is progressively impaired by the rising number of previous interventions at the bone site [27], only patients with a maximum of one previous operation at the non-union were selected for this study. Hence, our analysis did not show a correlation of non-union-healing associated either with previous non-operative fracture treatment or previous surgery (p = 0.329). Additionally, it is well documented that smokers are significantly more at risk to develop complicated fracture healing [1,10,27]. In the present study, there were only three smokers among the evaluated non-union patients: one smoker with 12 package years in the in the autologous-group and two smokers with a mean value of 25 package years in the allograft-group, respectively. Consequently, the low rate of smokers led to the low mean value of package years in each group, probably resulting in a lack of correlation between non-union-healing and smoking behavior (p = 0.176). In addition, the analysis did not show an association between the non-union-consolidation and patient's age (p = 0.312), sex (p = 0.242), BMI (p = 0.116), and ASA value (p = 0.576).

There are some possible disadvantages associated with the use of allografts. The first detriment is the additional cost for surgery compared to autologous bone grafting. However, Lohmann et al. evaluated recently the economic impact of ICABG in trauma surgery [28]. Mainly because of the extended operation time, harvesting was calculated to cost 213 €. In our study, all patients of the allograft-group were treated with the use of 2.5 cc Grafton^® ^Putty, which was announced in Osteotech's pricelist to cost 373 €. Thus, considering the economical aspect of both non-union procedures, DBM costs 160 € more compared to ICABG. The second handicap of allografts might be the potential immunogenicity compared to autologous grafting which was shown not only in animal models [29] but also probably in clinical applications [30-32]. Fortunately, we did not observe allograft-related immunological adverse reactions in our study but this potential problem should be considered using DBM.

Beside the disadvantages shown above, there are several benefits associated with the use of allografts. The doubtless advantages of DBM are first the unlimited availability, second the reduced operative time and bleeding, and third the avoidance of donor site complications which were documented in 20% of our study-patients treated by ICABG [8-15]. An additional advantage of DBM might be the beneficial effect in patients with more comorbidity: As documented in our trial, ASA values were significantly elevated in the allograft-group (p = 0.014). Despite this less advantageous cohort of patients treated with DBM, the incidence of non-union healing was comparable in both study groups. Based on these data we conclude that DBM is particularly advisable in morbid patients because of proved effectiveness in promoting non-union healing.

The limitations of the present study include first the small number of patients in each treatment group which is probably the result of a decreased rate of non-unions because of advanced therapy-options in acute fracture treatment [3]. Second, the investigator could not be blinded with regard to the donor-site scar at the iliac crest, thus follow-up data were not blindly assessed. Nevertheless, the research fellow was not the treating physician and therefore we do not think that lack of blinding influenced the findings of the study. Furthermore, since pain intensity and treatment-satisfaction (NRS) was self-assessed by patients and x-rays were evaluated by a study-independent radiologist a potential assessor-related bias could be minimized. Third, the non-prospective design and consequently a lack of randomization is a weakness of the study.

## Conclusion

In conclusion, we successfully incorporated augmentation with demineralized bone matrix allograft into a standard concept for the treatment of atrophic ununited extraarticular fractures of long bones in upper and lower extremities. Demineralized bone matrix proved to be equally effective as autologous bone graft in augmenting ORIF, since the healing incidence and time for consolidation of ununited fractures as well as pain intensity at follow-up was comparable in both groups. Nevertheless, autologous cancellous bone graft should still be considered as the gold standard in the treatment of non-unions, since at the present time first there is no better evidence available that supports the superiority of allografts and second allografting is more expensive. However, our study showed that patients treated by allograft-augmentation had no complications, reduced treatment-dissatisfaction, and a lack of donor site complications at the iliac crest region. Therefore we found our results adequately sound to conclude that the use of DBM should be offered to suitable patients in the preoperative consultation as a valuable alternative for autologous grafting. Moreover, we recommend non-union treatment with ORIF and augmentation with allografts first in morbid patients to reduce operative time and perioperative donor site complications, and second in patients with known osteopenia/osteoporosis and therefore limited availability of cancellous bone for autologous grafting. The low number of study patients, lack of randomization and the non-prospective study design weaken the power of this study. Hence, there is a need for further in-depth multicenter-investigations to verify not only our results but also to get further validated information about additional factors in order to optimize successful consolidation in long bone non-unions.

## Competing interests

The authors declare that they have no competing interests.

## Authors' contributions

OP designed the presented study, conducted the primary analysis and drafted the manuscript, assisted by SP and HT. AW performed the acquisition and analysis of data in cooperation with JZ, TL and BR. All authors have been involved in revising the manuscript and have given final approval of the version to be published.

## References

[B1] Calori GM Albisetti W Agus A Iori S Tagliabue L Risk factors contributing to fracture non-unions Injury 2007 38 Suppl 2 S11 18 10.1016/S0020-1383(07)80004-0 17920412

[B2] Giannoudis PV Einhorn TA Marsh D Fracture healing: the diamond concept Injury 2007 38 Suppl 4 S3 6 18224731 10.1016/S0020-1383(08)70003-2

[B3] Tzioupis C Giannoudis PV Prevalence of long-bone non-unions Injury 2007 38 Suppl 2 S3 9 10.1016/S0020-1383(07)80003-9 17920415

[B4] Einhorn TA Enhancement of fracture-healing J Bone Joint Surg Am 1995 77 940 956 7782368 10.2106/00004623-199506000-00016

[B5] Drosos GI Kazakos KI Kouzoumpasis P Verettas DA Safety and efficacy of commercially available demineralised bone matrix preparations: a critical review of clinical studies Injury 2007 4 S13 21 10.1016/S0020-1383(08)70005-6 18224733

[B6] Finkemeier CG Bone-grafting and bone-graft substitutes J Bone Joint Surg Am 2002 84-A 454 464 11886919 10.2106/00004623-200203000-00020

[B7] Keating JF McQueen MM Substitutes for autologous bone graft in orthopaedic trauma J Bone Joint Surg Br 2001 83 3 8 10.1302/0301-620X.83B1.11952 11245534

[B8] Arrington ED Smith WJ Chambers HG Bucknell AL Davino NA Complications of iliac crest bone graft harvesting Clin Orthop Relat Res 1996 300 309 10.1097/00003086-199608000-00037 8769465

[B9] Fernyhough JC Schimandle JJ Weigel MC Edwards CC Levine AM Chronic donor site pain complicating bone graft harvesting from the posterior iliac crest for spinal fusion Spine 1992 17 1474 1480 10.1097/00007632-199212000-00006 1471005

[B10] Kurz LT Garfin SR Booth RE Jr Harvesting autogenous iliac bone grafts Spine 1989 14 1324 1331 10.1097/00007632-198912000-00009 2617362

[B11] Robertson PA Wray AC Natural history of posterior iliac crest bone graft donation for spinal surgery: a prospective analysis of morbidity Spine 2001 26 1473 1476 10.1097/00007632-200107010-00018 11458153

[B12] Russell JL Block JE Surgical harvesting of bone graft from the ilium: point of view Med Hypotheses 2000 55 474 479 10.1054/mehy.2000.1095 11090293

[B13] Skaggs DL Samuelson MA Hale JM Kay RM Tolo VT Complications of posterior iliac crest bone grafting in spine surgery in children Spine 2000 25 2400 2402 10.1097/00007632-200009150-00021 10984795

[B14] Summers BN Eisenstein SM Donor site pain from the ilium J Bone Joint Surg Br 1989 71 677 680 2768321 10.1302/0301-620X.71B4.2768321

[B15] Younger EM Chapman MW Morbidity at bone graft donor sites J Orthop Trauma 1989 3 192 195 10.1097/00005131-198909000-00002 2809818

[B16] Williams A Szabo RM Bone transplantation Orthopedics 2004 27 488 495 quiz 496-487 15181946 10.3928/0147-7447-20040501-17

[B17] De Long WG Jr Einhorn TA Koval K McKee M Smith W Sanders R Watson T Bone grafts and bone graft substitutes in orthopaedic trauma surgery J Bone Joint Surg Am 2007 89 649 658 10.2106/JBJS.F.00465 17332116

[B18] Urist MR Bone: formation by autoinduction Science 1965 150 893 899 10.1126/science.150.3698.893 5319761

[B19] Hierholzer C Sama D Toro JB Peterson M Helfet DL Plate fixation of ununited humeral shaft fractures: effect of type of bone graft on healing J Bone Joint Surg Am 2006 88 1442 1447 10.2106/JBJS.E.00332 16818968

[B20] Ziran B Cheung S Smith W Westerheide K Comparative efficacy of 2 different demineralized bone matrix allografts in treating long-bone nonunions in heavy tobacco smokers Am J Orthop 2005 34 329 332 16130350

[B21] Wilkins RM Chimenti BT Rifkin RM Percutaneous treatment of long bone nonunions: the use of autologous bone marrow and allograft bone matrix Orthopedics 2003 26 s549 554 12755223 10.3928/0147-7447-20030502-04

[B22] Wilkins RM Kelly CM The effect of allomatrix injectable putty on the outcome of long bone applications Orthopedics 2003 26 s567 570 12755227 10.3928/0147-7447-20030502-08

[B23] Gustilo RB Anderson JT Prevention of infection in the treatment of one thousand and twenty-five open fractures of long bones: retrospective and prospective analyses J Bone Joint Surg Am 1976 58 453 458 773941

[B24] Iwata H Sakano S Itoh T Bauer TW Demineralized bone matrix and native bone morphogenetic protein in orthopaedic surgery Clin Orthop Relat Res 2002 99 109 10.1097/00003086-200202000-00010 11937869

[B25] Peterson B Whang PG Iglesias R Wang JC Lieberman JR Osteoinductivity of commercially available demineralized bone matrix J Bone Joint Surg Am 2004 86-A 2243 2250 15466734 10.2106/00004623-200410000-00016

[B26] Wang JC Alanay A Mark D Kanim LE Campbell PA Dawson EG Lieberman JR A comparison of commercially available demineralized bone matrix for spinal fusion Eur Spine J 2007 16 1233 1240 10.1007/s00586-006-0282-x 17205237 PMC2200779

[B27] Calori GM Phillips M Jeetle S Tagliabue L Giannoudis PV Classification of non-union: need for a new scoring system? Injury 2007 39 Suppl 2 S59 63 10.1016/S0020-1383(08)70016-0 18804575

[B28] Lohmann H Grass G Rangger C Mathiak G Economic impact of cancellous bone grafting in trauma surgery Arch Orthop Trauma Surg 2007 127 345 348 10.1007/s00402-006-0277-4 17294203

[B29] Bos GD Goldberg VM Zika JM Heiple KG Powell AE Immune responses of rats to frozen bone allografts J Bone Joint Surg Am 1983 65 239 246 6337163 10.2106/00004623-198365020-00015

[B30] Ziran BH Smith WR Morgan SJ Use of calcium-based demineralized bone matrix/allograft for nonunions and posttraumatic reconstruction of the appendicular skeleton: preliminary results and complications J Trauma 2007 63 1324 1328 10.1097/01.ta.0000240452.64138.b0 18212656

[B31] Friedlaender GE Horowitz MC Immune responses to osteochondral allografts: nature and significance Orthopedics 1992 15 1171 1175 1409127 10.3928/0147-7447-19921001-08

[B32] Horowitz MC Friedlaender GE Qian HY The immune response: the efferent arm Clin Orthop Relat Res 1996 25 34 10.1097/00003086-199605000-00004 8620649

